# Supplementary material for: Splice-Junction-Based Mapping of Alternative Isoforms in the Human Proteome
Source: Cell Rep. Author manuscript; Available in PMC 2020 Jan 15. (PMC6961840; doi:10.1016/j.celrep.2019.11.026)
Supplement: 2 [file NIHMS1546469-supplement-2.zip › DF1/PXD010154/Liver/Liver_14_LRCH4_VGGAAAVSTQAMHNLLKPGLR.pdf]

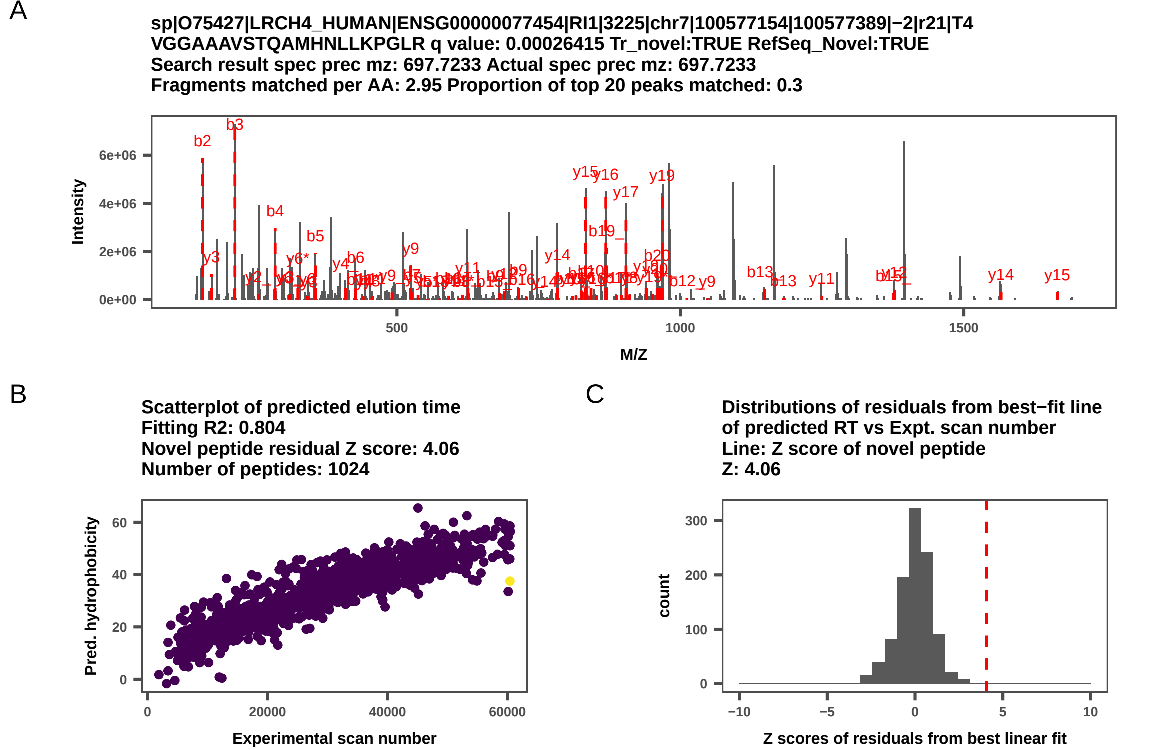
A
sp|O75427|LRCH4_HUMAN|ENSG00000077454|RI1|3225|chr7|100577154|100577389|−2|r21|T4
VGGAAAVSTQAMHNLLKPGLR q value: 0.00026415 Tr_novel:TRUE RefSeq_Novel:TRUE
Search result spec prec mz: 697.7233 Actual spec prec mz: 697.7233
Fragments matched per AA: 2.95 Proportion of top 20 peaks matched: 0.3
Intensity
6e+06
4e+06
2e+06
0e+00
b2
b3
y3
b4
b5
y6*
y4
b6
y9
y15
y16
y17
y19
b19
y14
b20
b13
y11
y14
y15
y12
b12
500
1000
1500
M/Z
B
Scatterplot of predicted elution time
Fitting R2: 0.804
Novel peptide residual Z score: 4.06
Number of peptides: 1024
Pred. hydrophobicity
60
40
20
0
0
20000
40000
60000
Experimental scan number
C
Distributions of residuals from best−fit line
of predicted RT vs Expt. scan number
Line: Z score of novel peptide
Z: 4.06
count
300
200
100
0
−10
−5
0
5
10
Z scores of residuals from best linear fit
